# Isolated knee extensor exercise training improves skeletal muscle vasodilation, blood flow, and functional capacity in patients with HFpEF

**DOI:** 10.14814/phy2.15419

**Published:** 2022-08-03

**Authors:** Christopher M. Hearon, Mitchel Samels, Katrin A. Dias, James P. MacNamara, Benjamin D. Levine, Satyam Sarma

**Affiliations:** ^1^ Institute for Exercise and Environmental Medicine Texas Health Presbyterian Hospital Dallas Dallas Texas USA; ^2^ University of Texas Southwestern Medical Center Department of Internal Medicine Dallas Texas USA

## Abstract

Patients with HFpEF experience severe exercise intolerance due in part to peripheral vascular and skeletal muscle impairments. Interventions targeting peripheral adaptations to exercise training may reverse vascular dysfunction, increase peripheral oxidative capacity, and improve functional capacity in HFpEF. Determine if 8 weeks of isolated knee extension exercise (KE) training will reverse vascular dysfunction, peripheral oxygen utilization, and exercise capacity in patients with HFpEF. Nine HFpEF patients (66 ± 5 years, 6 females) performed graded IKE exercise (5, 10, and 15 W) and maximal exercise testing (cycle ergometer) before and after IKE training (3x/week, 30 min/leg). Femoral blood flow (ultrasound) and leg vascular conductance (LVC; index of vasodilation) were measured during graded IKE exercise. Peak pulmonary oxygen uptake (V̇O_2_; Douglas bags) and cardiac output (Q_C_; acetylene rebreathe) were measured during graded maximal cycle exercise. IKE training improved LVC (pre: 810 ± 417, post: 1234 ± 347 ml/min/100 mmHg; *p* = 0.01) during 15 W IKE exercise and increased functional capacity by 13% (peak V̇O_2_ during cycle ergometry; pre:12.4 ± 5.2, post: 14.0 ± 6.0 ml/min/kg; *p* = 0.01). The improvement in peak V̇O_2_ was independent of changes in Q̇c (pre:12.7 ± 3.5, post: 13.2 ± 3.9 L/min; *p* = 0.26) and due primarily to increased a‐vO_2_ difference (pre: 10.3 ± 1.6, post: 11.0 ± 1.7; *p* = 0.02). IKE training improved vasodilation and functional capacity in patients with HFpEF. Exercise interventions aimed at increasing peripheral oxidative capacity may be effective therapeutic options for HFpEF patients.

## Graphical abstract

This study demonstrates residual vascular plasticity in patients with HFpEF. IKE training may be beneficial for patients with severe peripheral limitations to exercise capacity or dyspnea during traditional aerobic training.

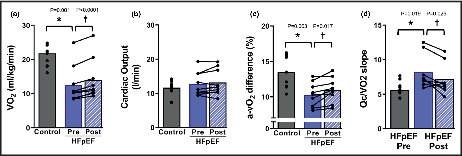

## 1 INTRODUCTION

The clinical manifestation of heart failure with preserved ejection fraction (HFpEF) is exercise intolerance, quantified as low‐peak aerobic power (peak V̇O_2_), and dyspnea on exertion that are associated with reduced quality of life (Kitzman et al., 2002). The mechanisms responsible for these impairments in functional capacity are multifactorial. Traditional aerobic exercise training interventions show some efficacy in improving peak V̇O_2_ in patients with HFpEF (Pandey et al., 2015). However, results from a larger clinical trial indicate that the benefit of generalized moderate or high‐intensity whole‐body exercise training may not be greater than regular guideline‐based physical activity (Mueller et al., 2021). Therefore, developing alternative exercise therapies that are well tolerated, pathophysiologically specific, and capable of enhancing the effect of exercise training is needed.

HFpEF patients have severe myocardial stiffness (Prasad et al., 2010), diastolic dysfunction, and poor cardiac reserve (Borlaug et al., 2006) that make the heart susceptible to large increases in pulmonary capillary wedge pressure during exercise. These central abnormalities are compounded by decrements in peripheral oxygen utilization due to impaired microvascular function (Lee, Barrett‐O'Keefe, Nelson, et al., 2016; Ratchford et al., 1985), O_2_ diffusive capacity (Houstis et al., 2018; Kitzman et al., 2014), and skeletal muscle myocyte oxidative capacity (Kitzman et al., 2014; Weiss et al., 2017; Zamani et al., 2021). While exercise training can improve peak V̇O_2_ in HFpEF patients, the majority (>80%) of improvement is due to adaptations in peripheral oxygen utilization (Haykowsky et al., 2012; Tucker et al., 2018). These findings indicate that peripheral vascular and skeletal muscle function may be key points of intervention in the oxygen transport cascade that are amenable to improvements with targeted exercise training.

Decrements in peripheral oxidative capacity can be targeted by utilizing small muscle mass exercise, typically of the knee extensors (isolated knee extensor exercise; IKE), to avoid central limitations to exercise performance (Andersen et al., 1985; Tyni‐Lenné et al., 1999). In heart failure with reduced ejection fraction, targeting peripheral adaptations with IKE improves whole‐body functional capacity due to residual vascular plasticity (convective transport) and enhanced diffusive O_2_ transport (Esposito et al., 2011, 2015). It is unclear whether HFpEF patients have similar vascular plasticity in response to IKE training. Therefore, we tested the hypothesis that 8 weeks of IKE training would reverse decrements in vascular function during knee extension exercise and improve functional capacity (peak V̇O_2_) independent of cardiac adaptations.

## 2 METHODS

### 2.1 Ethical statement

All subjects signed an informed consent form approved by the institutional review boards of the University of Texas Southwestern Medical Center and Presbyterian Hospital, Dallas, Texas (clinicaltrials.gov; NCT03465072).

### 2.2 Subject characteristics

A total of 12 patients (>65 years of age) with a clinical history of HF by Framingham criteria in addition to objective evidence for pulmonary congestion by chest radiograph or right heart catheterization and an ejection fraction >50% at the time of index hospitalization. Exclusion criteria included atrial fibrillation at the time of the study, recent (<1 year) myocardial infarction, prior coronary artery bypass graft, a history of surgical revascularization or multivessel percutaneous revascularization, stable angina, coronary artery disease with provocable ischemia, moderate to severe valvular heart disease, renal failure (creatinine >2.5 g/dl), chronic pulmonary disease, congenital heart disease, HF admission within the last 5 months, New York Heart Association HF class > III, and warfarin use. Three subjects were excluded due to vascular plaques that precluded ultrasound assessment of the common femoral artery (*n* = 2) or an inability to operate the knee ergometer (*n* = 1). Nine healthy, sedentary, senior controls were enrolled strictly to provide comparative baseline data. Controls completed a detailed medical history, examination, 12‐lead electrocardiogram, and stress exercise test with an echocardiogram to exclude coronary artery disease, valvular disease, and atrial flutter/fibrillation. Healthy control subjects were excluded if they had renal insufficiency and a history of nicotine use (past 10 years).

### 2.3 Study design

**FIGURE 1:**
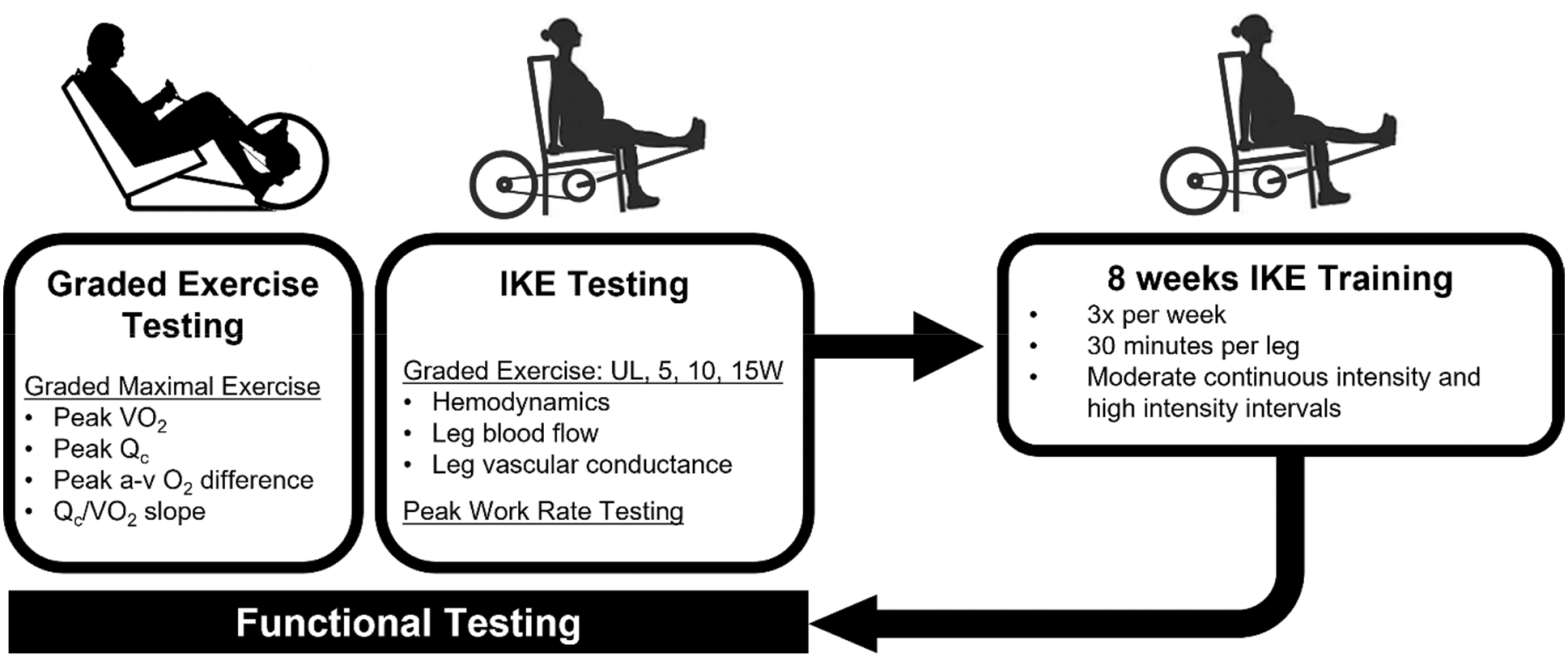
Experimental protocol. Patients with HFpEF (*n* = 9) completed graded cardiopulmonary exercise testing on a cycle ergometer to determine peak V̇O_2_, cardiac output (Q̇c) and a‐v O_2_ difference, and Q_c_/V̇O_2_ slope during maximal exercise. Patients also completed assessments of hemodynamics and a leg blood flow during isolated knee extension exercise to quantify vascular function during exercise independent of central cardiac limitations. Patients were then trained for 8 weeks using the IKE method to maximize peripheral adaptations to exercise. At the end of 8 weeks, graded exercise testing and IKE testing were repeated to determine the effect of IKE training on peripheral vascular function and whole‐body functional capacity. HFpEF, heart failure with preserved ejection fraction; IKE, isolated knee extension; UL, unloaded exercise.

Peak aerobic capacity and vascular function were assessed before and after 8 weeks of isolated knee extension (IKE) exercise training (Figure 1). Subjects performed IKE exercise training on a custom‐modified Monark ergometer. Briefly, the ankle of one leg was attached to the pedal of the cycle ergometer by a rigid bar. The ergometer was operated by rhythmic concentric contraction of the left or right quadriceps group with the leg returning passively to the starting position (90°) after each repetition as described previously (Andersen et al., 1985; Andersen & Saltin, 1985). Prior to training, each subject completed a graded maximal IKE test consisting of 1‐min stages and 5 W increments beginning at unloaded exercise and progressing until participants were unable to maintain 40 kicks per minute despite strong verbal encouragement. Training sessions were performed 3× per week for 8 weeks and consisted of 30 min of exercise per leg (1‐h total) at a kick rate of 30–50 kicks per minute. Exercise training was prescribed on an individual basis and increased in intensity throughout the intervention. Workouts initially consisted of steady state sessions (workloads that could be maintained for 30 min) and progressed to 8 × 2 min interval and 4 × 4 min interval sessions. The goal was to perform two interval sessions and one steady state session per week. Subjects performed maximal IKE testing intermittently to assess improvements in IKE capacity and adjust workloads accordingly.

### 2.4 Cardiopulmonary exercise testing

Each subject performed two graded exercise tests to determine peak V̇O_2_ at baseline. Each test was performed in a semi‐recumbent position on an electronically braked cycle ergometer with a pedal rate of ~60 rpm (10‐20 W increments every 2 min). The first test was used to familiarize subjects with experimental maneuvers and to screen for provocable ischemia by baseline and postexercise echocardiogram. The second exercise test occurred >72 h after the first test and consisted of two submaximal exercise bouts graded in intensity (SS1 and SS2) followed by a confirmatory graded maximal test. Following a minimum of 10 min of rest after submaximal exercise, peak exercise was assessed with the same incremental protocol as performed on Day 1. AV nodal blockers were withheld for 48 h prior to testing but other antihypertensive medications were continued.

Heart rate (HR) was monitored continuously via electrocardiogram (Schiller AT‐10; Welch Allyn Inc, Skaneateles Falls, New York, USA), and blood pressure was measured using electrosphygmomanometry (Suntech Tango+; Morrisville, North Carolina, USA). Measurements of ventilatory gas exchange were made on a breath‐by‐breath basis using an infrared turbine flow meter (VMM, Interface Associates; Laguna Niguel, California, USA) and mass spectrometry for rapid gas analysis via proprietary software. Breath‐by‐breath ventilatory gas exchange was verified at a steady state by the collection of expired gas in a Douglas bag for 1 min and analyzed by mass spectrometry. Steady‐state ventilatory volume was measured by use of a 120 L Tissot spirometer (W.E. Collins P‐1700; Braintree, Massachusetts, USA). Peak V̇O_2_ was defined as the highest oxygen uptake measured from at least a 30 s Douglas bag collection. Q̇c was measured using a modified acetylene rebreathing technique that is validated, reproducible, and equivalent to invasive measures of Q̇c during maximal exercise (Hardin et al., 2020). Arterial–venous oxygen difference (a‐vO_2_ difference) was calculated from the ratio between Q̇c and oxygen uptake using the Fick Equation (Hearon Jr. et al., 2019). Body mass and composition were measured by DEXA performed at Presbyterian Hospital Dallas (Lunar iDXA, GE Healthcare).

### 2.5 Leg blood flow

Leg blood flow was measured during rest, unloaded kicking (UL), 5, 10, and 15 W with a kick rate of 40 per minute, and the seat back of the isolated knee ergometer set to 60°. Each exercise stage was 3 min in duration, and a ~5‐min rest period was provided between the 10 and 15 W stage. Blood velocity and common femoral artery diameter were measured using Doppler ultrasonography (12 MHz linear array transducer, IE33, Phillips) 2–3 cm proximal to the bifurcation of the superficial and deep femoral arteries. Blood velocity was acquired with the sample volume centered within the vessel, optimized to span the vessel diameter without overlapping vessel walls, and set at an insonation angle of 60°. Blood velocity was determined by customized audio recording software that interfaced with the ultrasound to record forward and reverse Doppler frequencies continuously (Buck et al., 1985; Ely et al., 2017). Recordings were analyzed using an intensity‐weighted algorithm to determine mean blood velocity. Vessel diameters were acquired in triplicate at end‐diastole with a perpendicular insonation angle to optimize image resolution and analyzed using commercially available software (Xcelera, Phillips). Blood flow was calculated as described previously (Tymko et al., 2020) during the last 30 seconds of each workload. Leg vascular conductance (LVC, index of vasodilation) was calculated as (FBF/MAP) × 100.

### 2.6 Statistical analysis

This study was powered to detect a 1.5 ml/kg/min increase in peak V̇O_2_ with a standard deviation of 0.5 ml/kg/min, alpha 0.05, and power >80%. Hemodynamics and gas exchange variables at rest, submaximal, and peak exercise were compared independently within each exercise intensity by *t* tests. Comparisons between controls and HFpEF pre were made using unpaired *t* tests, and comparisons between HFpEF pre and HFpEF post were made with paired *t* tests. The effect of IKE training on hemodynamics and functional capacity during cycle exercise was assessed using paired *t* tests (HFpEF pre vs. HFpEF post). Data normality was determined using Shapiro–Wilk test.

The change in hemodynamics during the IKE protocol in control and HFpEF were compared using two‐way repeated measures ANOVA inclusive of group (Control, HFpEF pre, HFpEF post) × stage (Δ: UL, 5, 10, 15 W). Pairwise comparisons were made within the stage using Tukey's test which corrects for multiple comparisons (Graphpad 9). Significance was set a priori at *p* < 0.05. Data are expressed in text as mean ± SD unless noted otherwise.

## 3 RESULTS

Subject characteristics are presented in Table 1. Healthy controls were well matched for age, while HFpEF patients weighed more and had higher BMI and BSA.

**TABLE 1:** Subject characteristics

	Control	HFpEF pre
N (M:F)	9 (3:6)	9 (3:6)
Age (years)	68 (8)	67 (5)
Height (cm)	171 (8)	169 (9)
Weight (kg)	73 (12)	105 (13)*
BMI (kg/m^2^)	25 (3)	37 (6)*
BSA (m^2^)	1.86 (0.19)	2.22 (0.17)*
Peak IKE work rate	24 (7)	20 (15)
HF pharmacotherapy, *n* (%) pharmacotherapy		
ACEi or ARB	—	5 (55)
Aldosterone Antagonist	—	5 (55)
Aspirin	2 (22)	4 (44)
β‐blocker	—	4 (44)
Calcium channel antagonist	—	5 (55)
Diuretic	—	4 (44)
Statin	4 (44)	6 (66)
Insulin	—	3 (33)
Metformin	1 (11)	1 (11)

Abbreviations: BMI, body mass index; HF, heart failure; HFpEF, heart failure with preserved ejection fraction; IKE, Isolated Knee Extension.

* *p* < 0.05 vs. Control.

### 3.1 Vascular function during IKE exercise

**FIGURE 2:**
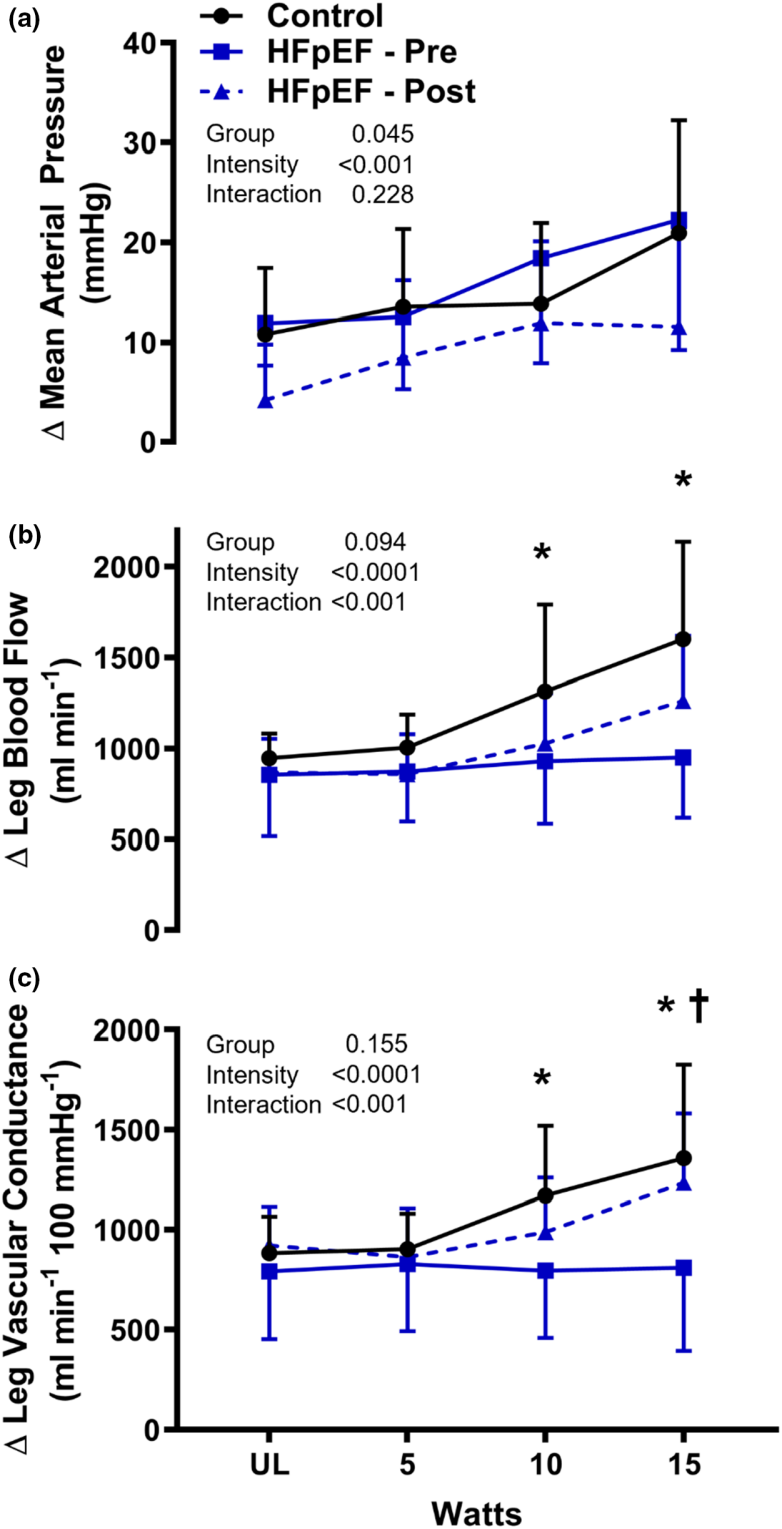
IKE training improves skeletal muscle vascular function in HFpEF. Change in (a) mean arterial pressure, (b) leg blood flow (femoral artery), and (c) vascular conductance during graded isolated knee extension exercise (IKE) in healthy, sedentary senior controls (*N* = 9), and HFpEF patients (*n* = 8) before and after 8 weeks of IKE training. HFpEF, heart failure with preserved ejection fraction; IKE, isolated knee extension. *HFpEF pre vs. Control; †HFpEF post vs. HFpEF pre.

In healthy controls, IKE exercise elicited a graded increase in LBF and LVC (Table 2). The leg blood flow response during IKE exercise was attenuated in HFpEF compared with control (ΔLBF at 15 W: Controls: 1601 ± 537, HFpEF pre: 950 ± 332 ml/min, *p* < 0.001) due to a blunted vasodilatory response (Δ LVC at 15 W: Controls: 1358 ± 469, HFpEF pre: 810 ± 417 ml/100 mmHg/min, *p* = 0.002) (Figure 2). Eight weeks of IKE training modestly improved peak IKE work rate (HFpEF pre: 20 ± 15, HFpEF post: 26 ± 12 W, *p* = 0.075). After training, the blood pressure response during IKE was significantly lower across exercise intensities (Figure 2; group effect *p* = 0.045). Despite lower systemic blood pressure, leg blood flow was maintained or increased due to improved vasodilation (Δ LVC at 15 W: HFpEF pre: 810 ± 417, HFpEF post: 1234 ± 347 ml/100 mmHg/min, *p* = 0.025) (Figure 2).

**TABLE 2:** Isolated knee extensor exercise hemodynamics

	Rest	UL	5 W	10 W	15 W
HR (bpm)					
Control	63 (8)	74 (9)	75 (9)	79 (10)	88 (13)
HFpEF pre	59 (9)	68 (11)	70 (12)	73 (12)	77 (14)
HFpEF post	61 (7)	67 (10)	68 (9)	71 (10)	78 (10)
MAP (mmHg) (l/min)					
Control	95 (11)	106 (11)	109 (10)	109 (13)	116 (15)
HFpEF pre	87 (8)	99 (8)	106 (12)	105 (12)	110 (13)
HFpEF post	88 (7)	93 (5)	97 (5)	100 (8)	100 (10)
LBF (ml/min)					
Control	173 (79)	1120 (145)	1179 (219)	1487 (538)	1774 (583)
HFpEF pre	335 (196)	1155 (327)	1194 (277)	1295 (369)	1334 (346)
HFpEF post	283 (94)	1151 (234)	1140 (235)	1309 (292)	1547 (383)
LVC (ml/100 mmHg/min)					
Control	180 (66)	1064 (157)	1085 (163)	1352 (371)	1538 (478)
HFpEF pre	379 (209)	1146 (297)	1177 (309)	1212 (345)	1221 (374)
HFpEF post	319 (98)	1241 (236)	1182 (247)	1308 (282)	1553 (375)

Abbreviations: HFpEF, heart failure with preserved ejection fraction; HR, heart rate; LBF, leg blood flow; LVC, leg vascular conductance; MAP, mean arterial pressure; UL, Unloaded.

### 3.2 Hemodynamics during submaximal cycle exercise

During submaximal exercise, controls and HFpEF patients exercised at a similar average workload (SS1: control: 24 ± 7, HFpEF: 20 ± 9 W, *p* = 0.34 and SS2: control: 44 ± 22, HFpEF: control 38 ± 21 W, *p* = 0.62) and showed no significant difference in absolute SS1 or SS2 V̇O_2_. The same submaximal workload was used for HFpEF patients pre and post IKE training. There was no consistent effect of IKE training on submaximal exercise hemodynamics in HFpEF (Table 3).

**TABLE 3:** Cycle exercise hemodynamics

	Rest	SS1	SS2	Peak
HR (bpm)				
Control	74 (14)	95 (10)	108 (12)	156 (7)
HFpEF pre	67 (11)	90 (9)	98 (6)*	122 (19)*
HFpEF post	65 (10)	88 (12)	94 (9)	123 (17)
MAP (mmHg)				
Control	95 (12)	107 (11)	111 (11)	125 (7)
HFpEF pre	84 (9)	104 (16)	101 (12)	108 (11)*
HFpEF post	85 (10)	101 (9)	102 (8)	111 (13)
Abs. V̇O_2_ (l/min)				
Control	0.21 (0.04)	0.69 (0.17)	0.90 (0.23)	1.51 (0.40)
HFpEF pre	0.31 (0.08)*	0.80 (0.19)	0.97 (0.30)	1.34 (0.53)
HFpEF post	0.31 (0.06)	0.80 (0.22)	0.93 (0.23)	1.47 (0.60)†
Rel. V̇O_2_ (ml/kg/min)				
Control	3.0 (0.4)	9.6 (1.5)	12.5 (1.9)	21.0 (3.2)
HFpEF pre	2.8 (0.5)	7.6 (1.3)*	9.2 (2.7)*	12.4 (5.2)*
HFpEF post	3.0 (0.5)	7.6 (1.4)	9.0 (2.1)	14.0 (6.0)†
Q̇_c_ (l/min)				
Control	3.77 (0.88)	7.11 (1.33)	8.36 (1.72)	11.20 (2.43)
HFpEF pre	4.59 (0.86)	8.96 (2.02)*	9.20 (1.90)	12.74 (3.52)
HFpEF post	5.33 (0.95)	8.97 (3.18)	9.6 (1.8)	13.17 (3.86)
SV (ml)				
Control	53 (15)	76 (15)	77 (14)	72 (17)
HFpEF pre	69 (10)*	98 (20)*	90 (20)*	104 (27)*
HFpEF post	83 (19)†	101 (28)	103 (22)†	105 (22)
a‐vO_2_ difference (%)				
Control	6.0 (2.0)	9.9 (2.2)	10.8 (1.5)	13.6 (2.3)
HFpEF pre	6.8 (1.3)	9.3 (1.1)	10.3 (1.6)	10.3 (1.6)*
HFpEF post	6.0 (1.3)†	9.3 (1.6)	9.6 (0.9)	11.0 (1.7)†
TPR (dyne/sec/cm^5^)				
Control	2120 (552)	1240 (305)	1106 (254)	937 (242)
HFpEF pre	1512 (346)*	966 (201)*	906 (203)	726 (164)*
HFpEF post	1305 (186)	971 (268)	871 (161)	723 (214)

Abbreviations: HFpEF, heart failure with preserved ejection fraction; HR, heart rate; MAP, mean arterial pressure; Q̇_c_, cardiac output; SV, stroke volume; TPR, total peripheral resistance; V̇O_2_, peak oxygen utilization.

* *p* < 0.05 vs. Control;

† *p* < 0.05 vs. HFpEF Pre. All comparisons are made within exercise intensity using unpaired (HFpEF pre vs. control) or paired (HFpEF post vs. HFpEF pre) *t* tests.

### 3.3 Peak cycle exercise hemodynamics and functional capacity

**FIGURE 3:**
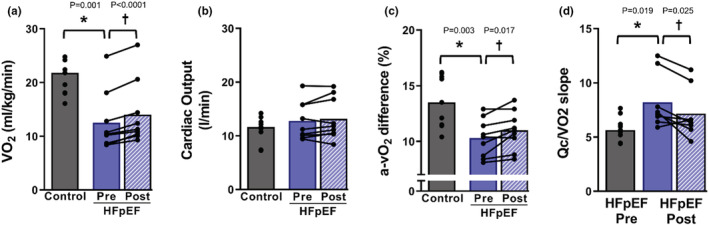
(a) IKE training improves functional capacity in HFpEF. V̇O_2_, (b) Cardiac Output, and (c) a‐vO_2_ difference during peak cycle exercise in healthy, sedentary, senior controls (*n* = 9), and HFpEF patients before and after 8 weeks of IKE training. (d) Q̇c/V̇O_2_ slope during graded cycle exercise testing in controls and HFpEF patients after 8 weeks of IKE training. HFpEF, heart failure with preserved ejection fraction; IKE, isolated knee extension. *HFpEF pre vs. Control; †HFpEF post vs. HFpEF pre.

At baseline, HFpEF patients had a similar absolute peak V̇O_2_ (Control: 1.51 ± 1.96, HFpEF pre:1.34 ± 0.53 L/min; *p* = 0.38) (Table 3) that was significantly lower when scaled to body mass (Control: 21.0 ± 3.2, HFpEF pre:12.4 ± 5.2 ml/kg/min; *p* = 0.001) (Figure 3). Peak Q̇c was not different between groups at baseline (control: 11.20 ± 2.43, HFpEF pre: 12.74 ± 3.52 L/min; *p* = 0.314), however, HFpEF patients had lower peak a‐vO_2_ difference (control: 13.6 ± 2.3, HFpEF pre: 10.3 ± 1.6%; *p* = 0.003) and an exaggerated rise in Q̇c relative to V̇O_2_ (Q̇c/V̇O_2_ slope: Control: 5.8 ± 1.1, HFpEF: 8.1 ± 2.4 A.U.; *p* = 0.019) (Figure 3). IKE training improved peak work rate (HFpEF pre: 82 ± 40, HFpEF post: 93 ± 46 W; *p* = 0.038), absolute V̇O_2_ (HFpEF Pre: 1.34 ± 0.53, HFpEF post: 1.47 ± 0.60 L/min; *p* = 0.006) and relative V̇O_2_ (HFpEF Pre: 12.4 ± 5.2, HFpEF post: 14.0 ± 6.0; *p* < 0.0001). The improvement in peak functional capacity was due to a greater a‐vO_2_ difference (HFpEF pre: 10.3 ± 1.6, HFpEF post: 11.0 ± 1.7%; *p* = 0.017) and was associated with a lower Q̇c/V̇O_2_ slope (HFpEF pre: 8.1 ± 2.4, HFpEF post: 7.2 ± 2.3 A.U.; *p* = 0.025). There was no change in peak Q̇c after IKE training (pre:12.7 ± 3.5, post: 13.2 ± 3.9; *p* = 0.26) (Figure 3) and IKE training had no effect on BMI (HFpEF pre: 37 ± 6, HFpEF post: 37 ± 6).

## 4 DISCUSSION

The primary findings of this investigation are that 8 weeks of IKE training in patients with HFpEF (1) restored the vasodilatory response to knee extension exercise, (2) increased aerobic capacity (cycle ergometry) by 13% (~1.5 ml/kg/min), and (3) reduced the hyperdynamic cardiac output response to exercise. The increase in peak functional capacity was due exclusively to increased peripheral oxygen extraction with no change in peak Q̇_C_. This is the first study to quantify residual skeletal muscle vascular plasticity in response to exercise training in HFpEF. The improvement in peak V̇O_2_ in the absence of central adaptations highlights the potential utility of exercise interventions that target peripheral vascular and skeletal muscle adaptations to improve overall functional capacity. The utility of IKE exercise may be especially important for subsets of HFpEF patients that suffer primarily from severe peripheral limitations to exercise capacity.

### 4.1 IKE training improves peripheral vascular function and hemodynamics in HFpEF

Vascular dysfunction in patients with HFpEF has been characterized primarily at the level of the conduit arterial circulation as it relates to vascular stiffness and dilatory function (e.g., FMD) (Kitzman & Haykowsky, 2016). While conduit arterial stiffness and vasodilatory dysfunction are common findings, they are not universal (Haykowsky et al., 2013; Hundley et al., 2007; Lee, Barrett‐O'Keefe, Garten, et al., 2016). Crucially, when normalizing for shear it appears conduit arterial function is relatively preserved in HFpEF compared with healthy controls (Lee, Barrett‐O'Keefe, Garten, et al., 2016). Further, a small number of studies have investigated the effect of exercise training on metrics of conduit arterial function and find that traditional exercise training modalities improve peak V̇O_2_ despite no change in indices of conduit arterial stiffness or function, which may be interpreted as a lack of vascular plasticity in HFpEF (Tucker et al., 2018). However, metrics of conduit arterial stiffness and vasodilatory function fail to quantify microvascular impairments that are most directly related to the regulation of blood flow during exercise. Along these lines, microvascular dysfunction is much more commonly observed in HFpEF (Balmain et al., 2007; Borlaug et al., 2010; Lee, Barrett‐O'Keefe, Garten, et al., 2016), though the effect of exercise training on microvascular function remains unclear.

Relatively, few studies have quantified the vasodilatory response to exercise in HFpEF patients. Previously, a series of investigations identified severe reductions in the leg and forearm vascular conductance during exercise in HFpEF that are not attributable to disease‐related decrements in cardiac or conduit arterial dysfunction (Lee, Barrett‐O'Keefe, Nelson, et al., 2016; Ratchford et al., 1985; Ratchford et al., 2019). These findings are in line with findings of microvascular dysfunction and skeletal muscle structural and oxidative deficiencies in HFpEF (Kitzman et al., 2014; Zamani et al., 2021). Our results confirm the presence of diminished skeletal muscle vasodilation during exercise in HFpEF patients (Lee, Barrett‐O'Keefe, Nelson, et al., 2016; Ratchford et al., 1985). Significant reductions in leg blood flow were evident even at low external workloads (10 W). Strikingly, blood flow plateaued and failed to increase even as exercise intensity continued to increase, confirming similar patterns observed in a prior investigation in HFpEF (Lee, Barrett‐O'Keefe, Nelson, et al., 2016). After 8 weeks of IKE, the vasodilatory response (vascular conductance) to exercise was restored to levels approaching healthy senior controls. While blood flow was also improved, IKE training did not restore blood flow fully to the levels measured in healthy controls. This failure to fully normalize blood flow may indicate that some metabolic adaptations, for example, changes in muscle fiber type, capillarity (Kitzman et al., 2014; Zamani et al., 2021), or mitochondrial function (Molina et al., 2016) that determine O_2_ demand and therefore blood flow, may be slower to improve in response to 8 weeks of IKE relative to vasodilatory responsiveness. Direct metrics of skeletal muscle structural and biochemical adaptations were not assessed in the current investigation, however, improvements in convective oxygen delivery alone are generally insufficient to improve oxidative capacity (Roca et al., 1992). Therefore, the improvement in V̇O_2_, a‐vO_2_ difference, and Q_C_/V̇O_2_ slope after IKE training likely reflects an associated improvement in muscle O_2_ diffusive capacity or skeletal muscle oxidative capacity. Prior work in HFrEF indicates residual plasticity in skeletal muscle diffusive capacity in response to 8 weeks of IKE training (Esposito et al., 2011, 2018; Magnusson et al., 1996). Future investigations are needed to characterize the improvement in skeletal muscle structure and function with IKE training in HFpEF.

In addition to improvements in vascular conductance and blood flow, 8 weeks of IKE training also reduced the pressor response to IKE in HFpEF patients to a level lower than that observed in healthy controls. Given the rapid (<2 weeks) onset of improvements in peak single‐leg work rate in response to IKE training observed in previous studies in HFrEF (Esposito et al., 2011), the improved neuromuscular function may be a key mechanism that lowers the level of central command required for the same absolute single‐leg work rate (lower relative rate) and, in turn, lowers blood pressure and left ventricular afterload (Mortensen et al., 2013). Previous studies indicate that inadequate blood flow is associated with impaired motor‐unit activation leading to excessive neuromuscular fatigue (Hammer et al., 2020). Similarly, neuromuscular fatigue related to detraining and poor blood flow is suggested to contribute to the perception of exercise intolerance in HFpEF (Weavil et al., 2021). Therefore, an improvement in blood flow and a reduction in neuromuscular fatigue is one potential mechanism contributing to the lower exercise pressor reflex after IKE training. Alternatively, improved vascular and skeletal muscle metabolic capacity may reduce afferent feedback components of the exercise pressor reflex (e.g., metaboreflex), though the contribution of this mechanism to exercise intolerance in HFpEF remains unclear.

Interestingly, the lower pressor response during IKE exercise after training (−10 mmHg) did not fully translate to a diminished pressor response during submaximal cycling exercise (−3 mmHg). This mirrors previous investigations showing the specificity of the heart rate and blood pressure training response to the type of exercise being performed (Kanstrup et al., 1991; Klausen et al., 1982; Saltin et al., 1976). However, most of these investigations of IKE training utilized short‐term (4–8 weeks) IKE training. It remains unclear whether longer duration IKE training would lead to a greater crossover of blood pressure and HR adaptations from IKE training to whole‐body exercise. Improved peripheral hemodynamics and central command could result in the improved reflex control of blood pressure and subsequently afterload during submaximal whole‐body exercise.

Overall, these data support previous observations that IKE training may have beneficial effects on neuromuscular and reflex autonomic control of the hemodynamic response to exercise. Given a sufficient exercise dose, adaptations to IKE training may translate to improved central hemodynamics during whole‐body exercise. This may be an important mechanism by which isolated small muscle mass training can improve whole‐body exercise tolerance independent of central hemodynamic adaptations.

### 4.2 IKE improves maximal exercise capacity

IKE training also improved maximal aerobic capacity quantified as peak V̇O_2_ during a traditional graded cycling exercise test. The improvement in aerobic capacity was due entirely to improved a‐vO_2_ difference, with no change in peak cardiac output. Importantly, the change in Q̇c for any given change in V̇O_2_ (Q̇c/V̇O_2_ slope) was lower after IKE training. Prior to IKE training HFpEF patients had an elevated Q̇c/V̇O_2_ slope, similar to what is observed in patients with skeletal muscle mitochondrial myopathies, where Q̇c increases disproportionately to metabolic demand (Bhella et al., 2011). Lower Q̇c/V̇O_2_ in response to training may be indicative of a more efficient distribution of blood flow to areas of highest metabolic demand, a reduction in previously overactive reflex autonomic control of cardiac output (e.g., central command, metaboreflex, mechanoreflex), or some combination of these factors.

The magnitude of the increase in peak V̇O_2_ (13%, ~1.5 ml/kg/min) is in line with the magnitude observed in previous exercise interventions utilizing traditional and high‐intensity whole‐body exercise training (Mueller et al., 2021; Pandey et al., 2015). Investigations in HFrEF patients show similar efficacy of IKE vs. traditional aerobic training, and some cases show greater efficacy of small muscle mass training compared with traditional exercise interventions (Esposito et al., 2011; Tyni‐Lenné et al., 1999). The results of the current investigation highlight the substantial plasticity in peripheral determinants of aerobic capacity and demonstrate that peripheral adaptations can translate to significant improvements in whole‐body functional capacity in the absence of cardiac plasticity. Finally, though the overall improvement in peak aerobic capacity with IKE is modest and of similar magnitude to traditional exercise training, IKE is less likely to produce symptoms of dyspnea and may minimize a primary barrier to exercise tolerance and adherence in HFpEF patients.

### 4.3 Clinical perspectives

Therapeutic interventions for HFpEF remain a major unmet need in cardiovascular science and medicine. The development of exercise interventions that improve exercise capacity while reducing the primary symptoms, including shortness of breath, remains a priority (Pandey & Kitzman, 2021). The mechanisms responsible for exercise intolerance in HFpEF are multifactorial and heterogeneous among patients, and limitations to oxygen transport and utilization exist along every step of the O_2_ transport cascade (Houstis et al., 2018). While central determinants of exercise intolerance are rarely reversed in response to exercise interventions, peripheral determinants of functional capacity retain the ability to adapt in response to exercise training. IKE, a training modality, that focuses on improving leg skeletal muscle and vascular function, not only improved peak work capacity, but also lowered the Q̇_C_/V̇O_2_ slope. Improved efficiency of blood delivery and utilization may result in the improved reflex control of heart rate and blood pressure, less blood returning to the right side of the heart for any given workload, and lower wedge pressure, though further investigations are needed to test this hypothesis. Ultimately, the adaptations to IKE training may be achieved while limiting shortness of breath during exercise training and has the potential to reduce symptoms of HFpEF and improve function during activities of daily living.

## 5 LIMITATIONS

The sample size in this investigation was sufficient to test the hypotheses presented in this manuscript as they relate to vascular function and functional capacity. Any outcomes that were not statistically significant should be interpreted with caution. While the relatively small sample size does preclude generalization of the efficacy of IKE to the clinical setting, it allowed for personalized monitoring and coaching during each exercise session and high‐resolution physiological phenotyping. This is important to demonstrate the physiological efficacy of IKE, but more work is needed to expand this approach to a wider population of patients that may encounter significant barriers to independent exercise adherence. An additional limitation is the lack of information regarding the structural and functional adaptation of the skeletal muscle in response to IKE training as well as metrics of leg‐specific diffusive and oxidative capacity. Future investigations are required to identify the specific skeletal muscle signaling pathways that retain plasticity in HFpEF patients and how they can be targeted to maximize the efficacy of exercise training.

## 6 CONCLUSIONS

IKE training reversed decrements in vascular function, improved peripheral oxygen utilization, and increased functional capacity in patients with HFpEF. The findings demonstrate significant residual plasticity in peripheral determinants of oxidative capacity and highlight the potential to improve exercise intolerance in HFpEF patients with exercise interventions designed to target peripheral adaptations. IKE training may be particularly beneficial for patients with primary peripheral limitations to exercise capacity or severe dyspnea during traditional aerobic training that limits exercise tolerance.

## AUTHOR CONTRIBUTIONS

Conception or design of work: CMH, BDL, and SS. Acquisition, analysis, or interpretation of data: CMH, MS, KAD, and JPM. Drafting and revision of the manuscript: CMH, MS, KAD, JPM, BDL, and SS. All authors approved the final version of the manuscript, agree to be accountable for all aspects of the work in ensuring that questions related to the accuracy or integrity of any part of the work are appropriately investigated and resolve, and all persons designated as authors qualify for authorship and those who qualify for authorship are listed.

## FUNDING INFORMATION

This work was supported by the National Institute of Health (grant nos. AG017479, 1P01HL137630, HL137285‐01, and 1P01HL137630). CMH was supported by the National Institute of Health (grant nos. F32HL137285 and K99HL153777).

## CONFLICT OF INTEREST

All data are available upon reasonable request. The authors have no competing interests. Experiments were performed at the Institute for Exercise and Environmental Medicine.
